# Nitric oxide induces cotyledon senescence involving co-operation of the *NES1*/*MAD1* and *EIN2*-associated *ORE1* signalling pathways in *Arabidopsis*

**DOI:** 10.1093/jxb/ert429

**Published:** 2013-12-12

**Authors:** Jing Du, Manli Li, Dongdong Kong, Lei Wang, Qiang Lv, Jinzheng Wang, Fang Bao, Qingqiu Gong, Jinchan Xia, Yikun He

**Affiliations:** ^1^College of Life Sciences, Capital Normal University, Beijing, 100048, PR China; ^2^Department of Cell Biology and Molecular Genetics, University of Maryland, College Park, MD 20742, USA; ^3^College of Life Sciences, Nankai University, Tianjin, 300071, PR China

**Keywords:** *Arabidopsis*, cotyledon, induced senescence, *NES1/MAD1*, nitric oxide, *ORE1/AtNAC2*.

## Abstract

After germination, cotyledons undertake the major role in supplying nutrients to the pre-photoautorophy angiosperm seedlings until they senesce. Like other senescence processes, cotyledon senescence is a programmed degenerative process. Nitric oxide can induce premature cotyledon senescence in *Arabidopsis thaliana*, yet the underlying mechanism remains elusive. A screen for genetic mutants identified the *nes1* mutant, in which cotyledon senescence was accelerated by nitric oxide. Map-based cloning revealed that *NES1* is allelic to a previously reported mitotic checkpoint family gene, *MAD1*. The *nes1*/*mad1* mutants were restored to the wild type, in response to nitric oxide, by transforming them with p*NES1*::*NES1*. Ectopic expression of *NES1* in the wild type delayed nitric oxide-mediated cotyledon senescence, confirming the repressive role of *NES1*. Moreover, two positive regulators of leaf senescence, the ethylene signalling component *EIN2* and the transcription factor *ORE1/AtNAC2/ANAC092*, were found to function during nitric oxide-induced senescence in cotyledons. The block of *ORE1* function delayed senescence and ectopic expression induced the process, revealing the positive role of *ORE1*. *EIN2* was required to induce *ORE1*. Furthermore, the genetic interaction analysis between *NES1* and *ORE1* showed that the *ore1* loss-of-function mutants were epistatic to *nes1*, suggesting the dominant role of *ORE1* and the antagonistic role of *NES1* during nitric oxide-induced cotyledon senescence in *Arabidopsis*.

## Summary

The *NES1/MAD1* gene acts antagonistically with the *EIN2*-associated *ORE1* signalling pathway to modulate the nitric oxide-induced *Arabidopsis* cotyledon senescence

## Introduction

Cotyledons are formed during embryogenesis. In most plants, the function of the cotyledon is to provide nutrients for seedling establishment. During seedling development, the cotyledon is initially heterotrophic, then becomes photosynthetic, and eventually senesces. As an integral part of development, the senescence of cotyledons is a process that leads to nutrient recycling and ends in cell death, and is accompanied by colour changes, the dismantling of chloroplasts, and the degradation of DNA, RNA, and protein (Krul, 1974; Peterman and Siedow, 1985). Phytohormones such as cytokinin and ethylene affect cotyledon senescence, with cytokinin preventing chlorophyll breakdown and ethylene initiating the onset of the senescence process (Ananieva *et al.*, 2008*a*; Jing *et al.*, 2008). Although cotyledon senescence has been studied for decades (McKersie *et al.*, 1987; Rukes and Mulkey, 1993), its underlying regulatory network is unclear.

In contrast, leaf senescence is better understood (Lim *et al.*, 2007). Based on transcriptome analysis, during natural leaf senescence, ~6–12% of *Arabidopsis* genes change expression (Buchanan-Wollaston *et al.*, 2005; Breeze *et al.*, 2011), which include >800 *SAG*s (senescence-associated genes). A number of *SAG*s have been well studied and established as markers. The favoured marker for monitoring age-dependent senescence is *SAG12*, whereas *SAG13* and *SAG14* are preferred for monitoring stress-induced senescence (Schippers *et al.*, 2007). Nevertheless, deletion or overexpression of many individual SAGs affect senescence to a limited extent, although there are a few exceptions (Seo *et al.*, 2011; Zhang and Gan, 2012), indicating the robust nature of the regulatory network. Certain transcription factors have been identified as positive regulators of age-dependent senescence in *Arabidopsis* by means of the loss-of-function mutant experiencing delayed leaf senescence, whereas others have been identified as negative regulators, in this case based on accelerated senescence in the loss-of-function mutant.

The better known positive regulators of leaf senescence are from the NAC (NAM, ATAF, and CUC) family. So far, a few have been well characterized, including *AtNAP* (*Arabidopsis* NAC domain containing protein 29) and *ORE1*/*AtNAC2*/*ANAC092* (ORESARA1). Not only does a block of function delay senescence, but ectopic expression induces early senescence (Guo and Gan, 2006; Rauf *et al.*, 2013). The control of the *ORE1* transcript involves *miR164* (microRNA164), which interacts with *ORE1* mRNA to trigger its degradation. *EIN2* (ethylene insensitive 2) and its downstream component *EIN3* of the ethylene signalling pathway negatively block *miRNA164* expression in an age-dependent manner, through the direct binding of *EIN3* to the promoter of *miRNA164*, which allows *ORE1* mRNA to accumulate (Kim *et al.*, 2009; Li *et al.*, 2013).

In addition to developmental senescence, various environmental stresses can induce or accelerate senescence. These environmental stresses may be biotic. Many of the stresses, including pathogen infection, drought, salinity, and extreme temperature (Bouchard and Yamasaki, 2008; Ma *et al.*, 2008; Neill *et al.*, 2008; Corpas *et al.*, 2009; Zhao *et al.*, 2009; Xuan *et al.*, 2010), are known to increase the production of nitric oxide in plants. A bioactive gas, nitric oxide has been suggested to be a signalling component that mediates stress responses (Arasimowicz and Floryszak-Wieczorek, 2007). Under certain conditions, nitric oxide is able to interact with ethylene and cytokinin. In tobacco, ethylene accumulation in response to ozone treatment depends on nitric oxide (Ederli *et al.*, 2006). In *Arabidopsis*, exposure to a high concentration of nitric oxide (48 ppm) results in ethylene accumulation (Magalhaes *et al.*, 2000). Nitric oxide may directly interact with cytokinin *in vivo* (Liu *et al.*, 2013), suggesting that nitric oxide represses endogenous cytokinin to some extent. In addition, nitric oxide represses the phosphorylation of the cytokinin signalling components AHP1 (histidine phosphotransfer protein 1) and ARR1 (response regulator 1) through the *S*-nitrosylation of AHP1, revealing the inhibitory effect of nitric oxide on cytokinin signalling (Feng *et al.*, 2013). Inducing the burst of nitric oxide during environmental stress-triggered senescence processes may play a critical role in modulating the levels of ethylene and cytokinin and the related pathways, thereby inducing senescence.

The sixth rosette leaf is a favoured plant part in leaf senescence studies on *Arabidopsis*. This leaf naturally starts to turn yellow ~21 d after its initiation, and completes senescence by about day 30. A similar phenotype has been observed in cotyledons, and a majority of *Arabidopsis* cotyledons finish senescing within 28 d (Smith, 2001). For stress-induced senescence studies in cotyledons, seedlings 5 d after germination are chosen for treatment (Weaver and Amasina, 2001). To study nitric oxide-regulated cotyledon senescence, the nitric oxide donor SNP (sodium nitroprusside) was mixed into agar and this was added to the cover of the Petri plates used for the 5 d seedling treatment. As nitroprusside breaks down, the seedlings are exposed to nitric oxide. Due to its volatility, the nitric oxide is able to diffuse though the air to reach the seedlings and prevents them from being exposed to the breakdown products aquapentacyanoferrate [Fe(CN)_5_·H_2_O]^3–^ and cyanide CN^–^ (Frank *et al.*, 1976; Arnold *et al.*, 1984), which are confined to the liquid phase in the Petri plate cover. After 3 d of treatment, senescence in cotyledons started to become visible and a screen was carried out for mutants that were more sensitive to nitric oxide, with respect to early cotyledon senescence. Here, a molecular genetic analysis of *NES1* (nitric oxide-induced early cotyledon senescence), which has been shown to be a negative regulator of nitric oxide-induced cotyledon senescence, is reported.

## Materials and methods

### Plant materials and treatment

All of the *Arabidopsis* mutagenic seeds, T-DNA insertion mutants, and transgenic plants used in this study were in a Col-0 (Columbia) background. For map-based cloning analysis, L*er* (Landsberg erecta) was used as the pollen acceptor and the *nes1-6* mutant was used as the pollen donor to create the F_1_ and then the F_2_ mapping populations. Five T-DNA insertional *NES1*-deficient mutants, *nes1-1* (SALK_080570), *nes1-2* (SALK_023425), *nes1-3* (SALK_073889), *nes1-4* (SALK_039008), and *nes1-5* (SALK_130471), and the *ORE1* deficient/overexpression lines *ore1* (SALK_090154) and 35S::*ORE1* (CS23887) were obtained and isolated from the ABRC (Arabidopsis Biological Resource Center). Before double mutant construction, *nes1-2* was backcrossed three times to the Col-0 background. All plants were grown in a controlled growth chamber at 21–22 °C under cool-white fluorescent light (80–100 μmol m^–2^ s^–1^) in a long-day photoperiod (16h light/8h dark).

Five-day-old seedlings, grown on agar plates with half-strength Murashige and Skoog (1/2 MS) medium supplemented with 0.6% (w/v) sucrose and 0.7% (w/v) agar, were used for the treatments with SNP, a chemical donor of nitric oxide when exposed to light, which was mixed in 10ml of 1% (w/v) agar medium and added only on the inside cover of Petri dishes.

### Measurements of chlorophyll content and fluorescence

Samples were taken and weighed at the indicated times, then placed into 5ml of 90% (v/v) acetone for extraction. The chlorophyll content of each sample was assayed by measuring the absorbance at 652, 665, and 750nm using a spectrophotometer. For fluorescence measurement, samples were taken every 30min after 20 μM SNP application, with 30min dark incubation before measurement at room temperature. The *F*_v_/*F*_m_ ratio was measured by MAXI Version (IMAGING-PAM *M-Series*, Germany) as described previously (Wingler *et al.*, 2004).

### Electrolyte leakage

For electrolyte leakage, 5-day-old seedlings were first treated with 20 μM nitroprusside for 24h. The excised cotyledons were then incubated in Milli-Q water and the conductivity of the solution was assayed with a conductivity cell (DJS-1C, GongGongPin Co., China). Finally, the samples were boiled for 5min and a second reading was taken. Electrolyte leakage is expressed as a percentage of the maximum.

### Gene expression analysis by RT-PCR

The mRNA levels were measured by quantitative real-time PCR (qRT-PCR). Total RNA was extracted from the seedlings using Trizol Reagent (Invitrogen, Gaithersburg, MD, USA) according to the manufacturer’s instructions. DNase was used to purify the RNA samples. The overall quality of the total RNA was monitored by formaldehyde–RNA denaturing electrophoresis and the *A*_260_/_280_ ratio (~2.0) using a NanoDrop Instrument spectrophotometer (NanoDrop Technologies, Wilmington, DE, USA). The concentration of total RNA was recorded. First-strand cDNA was synthesized with 2 μg of total RNA using M-MLV reverse transcriptase and an oligo(dT)_15_ primer (Promega) in a 20 μl mixture. The reaction was then diluted 2-fold with water and the cDNA was used as a template for both semi-quantitative RT-PCR and qRT-PCR. For the semi-quantitative PCR analysis, 1 μl of each reverse transcription reaction was used per PCR in a final volume of 20 μl. Semi-quantitative RT-PCR was performed by gene-specific primers under controlled conditions (Supplementary Table S2 available at *JXB* online). The reactions were repeated three times. Ubiquitin primers (*AtUBQ*) were used as the standard (Li *et al.*, 2007). Real-time PCR was performed using the SYBR green PCR master mix and the Bio-Rad iQ5 real-time PCR detection system. *ACT2* was used as the standard (Czechowski *et al.*, 2005). The primer sequences are given in Supplementary Table S3. All reactions were repeated at least three times. Statistical analysis of the results of real-time PCR was performed using iQ5 software.

### GUS staining

Seedlings were incubated with 10mM 5-bromo-4-chloro-indolyl-β-d-glucuronide (X-Glu) in 100mM phosphate buffer (pH 7.0) at 37 °C overnight. Chlorophyll was removed from leaves by clarification with 70% ethanol before observation. The whole β-glucuronidase (GUS) staining process was carried out according to the protocol of Jefferson (1987).

### Plasmid constructions and plant transformation

Specific primers, designed upstream of the open reading frame, were p*NES1*-EcoRI-F (5′-CCGGAATTCCGTCTTGCCAAAAA GCCAT-3′) and p*NES1*-BamHI-R (5′-GCGGGATCCGTCTGCG TCGAGAAATTAGGG-3′). The length of the cloned promoter was ~1100bp and included *Eco*RI and *Bam*HI restriction sites, as shown by the underlining. To obtain the *NES1* gene, primers were designed at both ends of the open reading frame. The primers were *NES1*-BamHI-F (5′-CGCGGATCCATGATTTTGAGAACTCCG-3′) and *NES1*- NcoI-R (5′-GTACCCATGGGATATAGCGTCCGACGGTTG-3′). The cloned CDS (coding sequence) (~2200bp) was amplified from cDNA, including *Bam*HI and *Nco*I enzyme sites. The promoter fragment was separately recombined into pCAMBIA1301 and pCAMBIA1302 to obtain *NES1*::GUS and p*NES1*::GFP (green fluorescent protein). The CDS of the *NES1* gene was then recombined into p*NES1*::GFP to obtain p*NES1*::*NES1*::GFP. To obtain p*NES1*::*NES1* and 35S::*NES1*, the primers for the CDS were modified through replacement of the restriction sites, which were *NES1*-NcoI-F (5′-GTACCCATGGATGATTTTGAGAACTCCG-3′) and *NES1*-NheI-R (5′-GATGCTAGCTCAATATAGCGTCCGAC-3′).

Transgenic lines were generated using the *Agrobacterium tumefaciens* LBA4404 vacuum infiltration method. Seeds of the first-generation transgenic line T_1_ from infiltrated plants were germinated on 1/2 MS medium containing 25mg l^–1^ hygromycin B. Several lines were obtained for each transformation and at least three generations of resistance screening were performed for preparation of material.

### Western blot

Samples of cotyledons were collected and ground in liquid nitrogen, and then incubated with an extraction buffer [0.1M TRIS-HCl, pH 8.3, 5mM dithiothreitol (DTT), 5mM EDTA, and protease inhibitor]. The Bradford protein assay was used for quantification and normalization. Proteins were resolved under reducing conditions by using 10% SDS–polyacrylamide gels. The proteins were transferred onto ployvinylidene difluoride (PVDF) membranes (Immobilon-P from Millipore), which were incubated separately with a primary GFP antibody (Roche, diluted 1:5000) and then a secondary peroxidase-conjugated anti-mouse antibody (Santa Cruz, diluted 1:10 000) for 2h at room temperature in TBS (20mM TRIS-HCl, pH 7.8, 180mM NaCl) supplemented with 4% (w/v) skimmed milk powder. After incubation, the membranes were washed twice (10min each) with TBS containing 0.05% Tween-20. After the final wash, membrane-associated peroxidase activity was visualized by using the ECL kit (Amersham Pharmacia).

## Results

### Map-based cloning

**Fig. 1.**
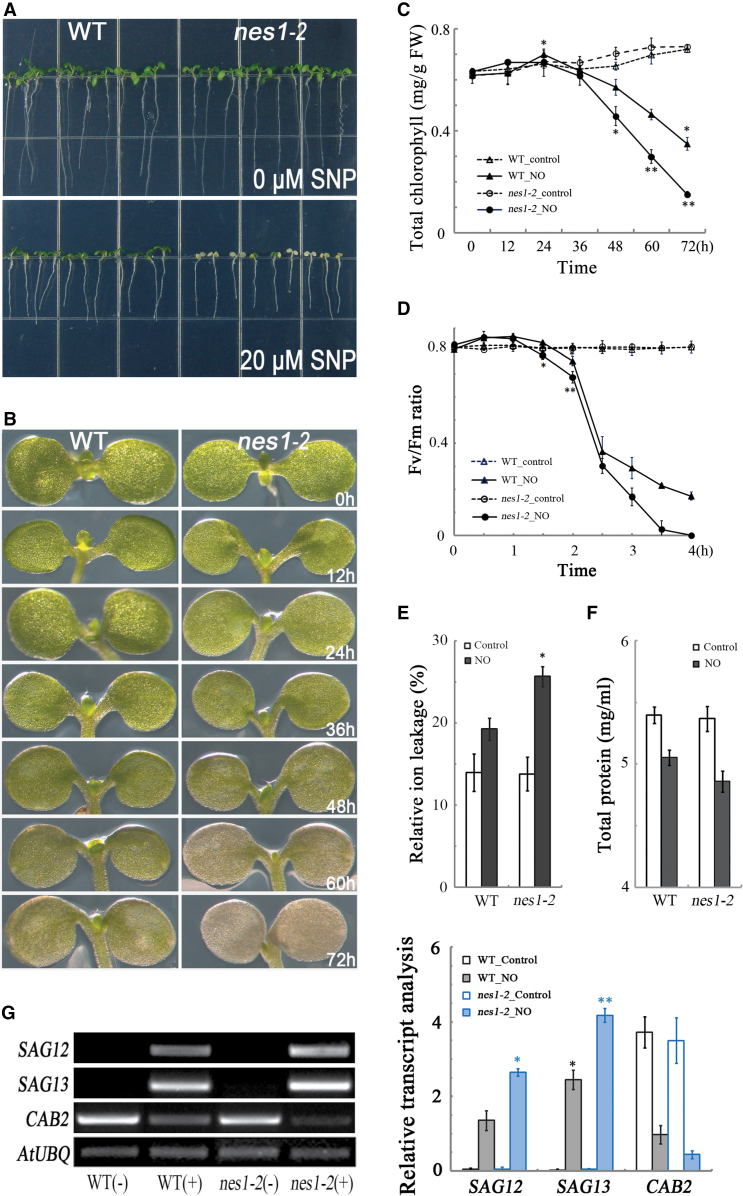
Nitric oxide-induced cotyledon senescence is accelerated in *nes1-2*. (A) Phenotypic comparison of 5-day-old wild type (WT) and *nes1-2* treated as indicated for 72h. (B) Higher magnification images of plants treated as in (A) with 20 μM nitroprusside. (C) Chlorophyll content as a function of time for plants treated as in (A). Data are the means of 20 seedlings ±SE with four replicates for each time point. (D) Chlorophyll fluorescence parameter (*F*_v_/*F*_m_) for plants treated with nitric oxide. Data are means of five seedlings ±SE, with four replicates for each time point. (E) Protein content of plans treated as in (A) with 20 μM nitroprusside for 24h. (F) Electrolyte leakage of plants treated as in (E). Data are means of five seedlings ±SE, with three replicates for each time point. (G) Semi-quantitative RT-PCR analysis of the indicated genes for plants treated as in (E). The intensity of bands was statistically analysed. Data are mean values ±SE, with three replicates for each sample. Asterisks indicate significant differences (*P < 0.05; **P ≤ 0.01).

Chemically mutagenized, light-grown seedlings were screened for enhanced cotyledon senescence in response to treatment with nitric oxide, and a line, named *nes1-6*, was isolated. With exposure to 20 μM SNP, cotyledon senescence in the mutant was markedly enhanced compared with the wild type (Fig. 1A, B). Ferricyanide was used as a control to exclude the side effects of the SNP breakdown products. In this condition, the two genotypes showed little difference from the non-treated seedlings (Supplementary Fig. S2 at *JXB* online), confirming the accelerated cotyledon senescence was caused by gaseous nitric oxide.

Based on a mapping population from a cross between the mutant *nes1-6* (Col-0) and the wild type (L*er*), the location of *NES1* was narrowed down to an interval (~20kb) between markers CER435084 and CER435911 (Supplementary Fig. S3A at *JXB* online), which contained three genes. T-DNA insertion mutants were all obtained, but only At5g49880 had a similar phenotype. For this locus, five T-DNA insertion alleles were obtained (Supplementary Fig. S3B) in which transcripts of At5g49880 were all detectable, except that there was sharply reduced abundance of *nes1-2* and *nes1-3* (Supplementary Fig. S3D). Although these five alleles showed enhanced cotyledon senescence under nitric oxide, *nes1-2* and *nes1-3* were the two strongest alleles. The insertion site of *nes1-2* was 125bp upstream of the start codon, in the same orientation as the gene. The site for *nes1-3* was in the eighth exon, 2511bp downstream of the start codon, in the opposite direction.

Genetic analysis of the originally chemically mutagenized allele, *nes1-6*, revealed that this allele was recessive (Supplementary Table S1 at *JXB* online). Analyses of F_1_ progeny from crosses between *nes1-6* and either *nes1-2* or *nes1-3* revealed similar accelerated cotyledon senescence to *nes1-6* (Supplementary Fig. S3C), confirming that *nes1-6* and the other five T-DNA insertion mutants were *nes1* lines. Finally, the nitric oxide-enhanced cotyledon senescence phenotype in *nes1-6* and *nes1-2* was rescued by transforming either line with the full-length At5g49880 coding sequence driven by its native promoter (Fig. 3D). Taken together, it is concluded that the nitric oxide-induced early cotyledon senescence in the *nes1* lines is caused by mutations in At5g49880.

At5g49880 has been previously annotated as a spindle assembly checkpoint protein MAD1 (mitotic arrest deficient 1). Interestingly, a role in cell cycle control was established for the closely related protein MAD2, but loss-of-function mitotic arrest phenotypes for *mad1* were not reported (Ding *et al.*, 2012). It appears that this gene has acquired a function in the pathway regulating senescence.

### Mutant phenotype

Because of its strong phenotype, the T-DNA insertion mutant *nes1-2/mad1* was used for further analysis. To establish a suitable timing for the nitric oxide treatment, cotyledon growth was first characterized (Supplementary Fig. S1A at *JXB* online). Cotyledon expansion was sustained and roughly isotropic for the first 10 d, and full size was attained a few days later (Supplementary Fig. S1B). The *nes1-2* line behaved similarly. To minimize interference from true leaves, which emerged on day 5, day 5 after germination was chosen as the time for the nitric oxide treatment.

A typical characteristic of senescence, whether developmental or stress induced, is the loss of chlorophyll. Treating 5-day-old seedlings with 20 μM nitroprusside for 72h caused the cotyledons to become slightly bleached in the wild type but substantially more so in the mutant (Fig. 1A). A time course showed that the cotyledons in the mutant were visibly less green by 60h of treatment, and to an even greater extent after 72h (Fig. 1B). This visual impression was confirmed by measuring the chlorophyll content (Fig. 1C), which illustrated that the mutant had started to lose chlorophyll by 48h of treatment and showed that chlorophyll loss appeared to be accelerated in the mutant by 60h compared with the wild type. To examine the photosynthetic apparatus with greater temporal precision, chlorophyll fluorescence was used to report the *F*_v_/*F*_m_ parameter. At time zero, this parameter was indistinguishable in the two genotypes and, interestingly, increased by ~10% over the first hour of treatment (Fig. 1B). After that, the *F*_v_/*F*_m_ ratio decreased steeply and after 2.5h had decreased significantly more in the mutant than in the wild type, indicating that the senescence programme needed only a few hours to be induced and that it happened more rapidly in the mutant.

In addition to losing chlorophyll, progression of senescence is determined by measuring other senescence parameters, such as loss of total protein content and increase of ion leakage, associated with cell death. Like leaves, cotyledons treated with nitric oxide to induce senescence also lost protein by 24h and the decrease was larger in the mutant than in the wild type (Fig. 1F). Likewise, ion leakage was induced and the extent of the leakage was significantly greater in the mutant (Fig. 1E).

To assay senescence on a molecular level, the expression of two *SAG*s, *SAG12* encoding a cysteine protease and *SAG13* encoding an alcohol dehydrogenase, both of which were up-regulated during senescence, was examined together with the expression of a photosynthetic gene, *CAB2* (chlorophyll *a*/*b*-binding protein 2), which was down-regulated. In the absence of treatment, both *SAG* transcripts were hardly detectable but *CAB2* was expressed strongly. The two genotypes appeared to be indistinguishable. However, with nitric oxide for 24h, the up-regulation of *SAG12* and *SAG13* was significantly stronger in *nes1-2*. The down-regulation of *CAB2* was apparent in the wild type and even more so in the mutant (Fig. 1G). Thus, taken together, *nes1-2* accelerates the progression of nitric oxide-induced senescence in cotyledons.

### Expression pattern of NES1

**Fig. 2.**
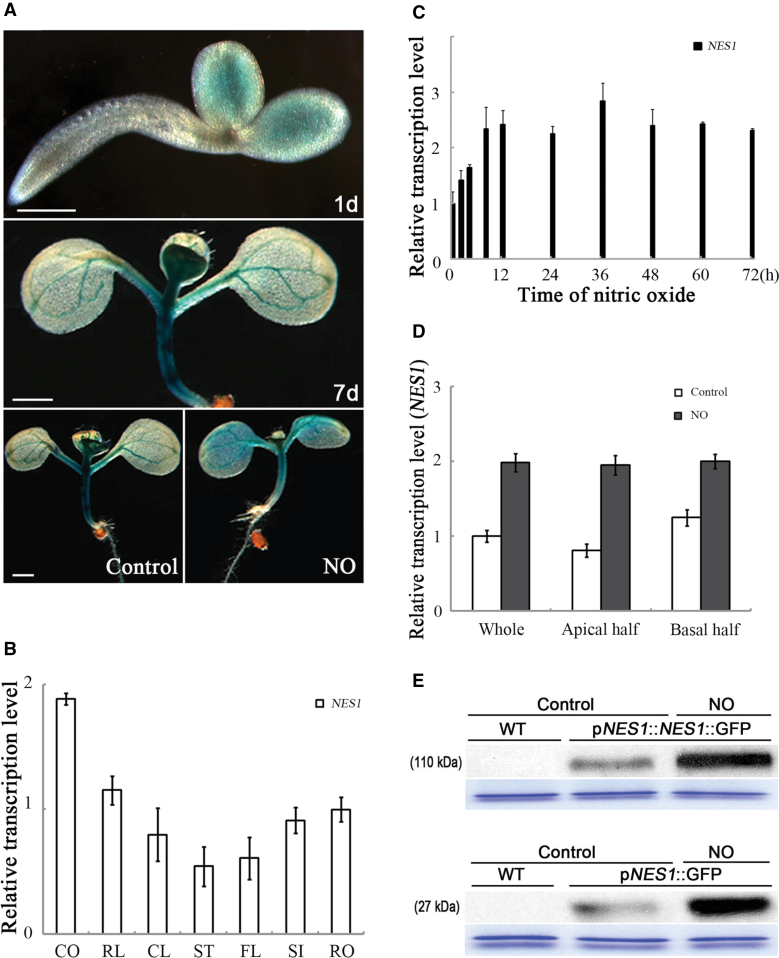
Expression of *NES1*. (A) GUS staining of transgenic seedlings carrying *pNES1*::GUS at the indicated times. The two lower panels show 5-day-old seedlings treated or not with 20 μM nitroprusside for 48h. Bars=100 μm (top) and 1mm (middle and bottom). (B) Organ-specific expression of *NES1*, with quantitative real-time PCR. CO, cotyledons; RL, rosette leaves; CL, cauline leaves; ST, stems; FL, flowers; SI, siliques; RO, roots. (C) The *NES1* transcript abundance as a function of treatment time. Seedlings treated as in Fig. 1B. Transcript quantified as in (B). (D) The *NES1* expression in apical and basal halves of the cotyledon. Seedlings treated as in Fig. 1E. (E) Western blot. Blots were probed with an anti-GFP primary antibody. Seedlings treated as for Fig. 1E. The loading control shows a band at ~50kDa stained with Coomassie blue.

To examine the expression of *NES1*, transgenic plants carrying a native *NES1* promoter–GUS gene fusion were first prepared. On the first day after germination, GUS activity was mainly detected in cotyledons, and, on the seventh day, GUS was more widely expressed throughout the seedling and concentrated in the vasculature (Fig. 2A). The GUS expression was strongly increased when 5-day-old seedlings were treated with 20 μM nitroprusside for 2 d. To examine the expression of *NES1* further, quantitative real-time RT-PCR was used, which allowed the detection of the native message. *NES1* was expressed in all organs, although the relative transcript levels were slightly higher in the cotyledons (1.88), compared with slightly lower levels in the stems (0.54) and the flowers (0.61), than the standard in the roots of 1.00 (Fig. 2B). When 5-day-old seedlings were treated with 20 μM nitroprusside, the message level of *NES1* in the cotyledons rose linearly within 12h and then remained steady up to at least 72h (Fig. 2C). When 5-day-old cotyledons were bisected into apical and basal halves, the latter had a slightly higher level of *NES1* basic expression. However, after a 24h treatment with 20 μM nitroprusside, the expression was raised to essentially the same level in both regions (Fig. 2D). Finally, to extend the analysis of expression to the protein level, a translational fusion was made between NES1 and GFP, driven by the *NES1* promoter (*pNES1*::*NES1*::GFP). As a control, GFP alone driven by the *NES1* promoter (*pNES1::*GFP) was used. Based on probing a western blot with anti-GFP antibody, the constructs were expressed in untreated seedlings, and both were strongly up-regulated by treatment with nitroprusside, with no detectable signal in the wild-type control (Fig. 2E). Taking these results together, NES1 appears to be widely and constitutively expressed and strongly up-regulated by nitric oxide.

### *NES1* as a negative regulator of nitric oxide-induced cotyledon senescence

**Fig. 3.**
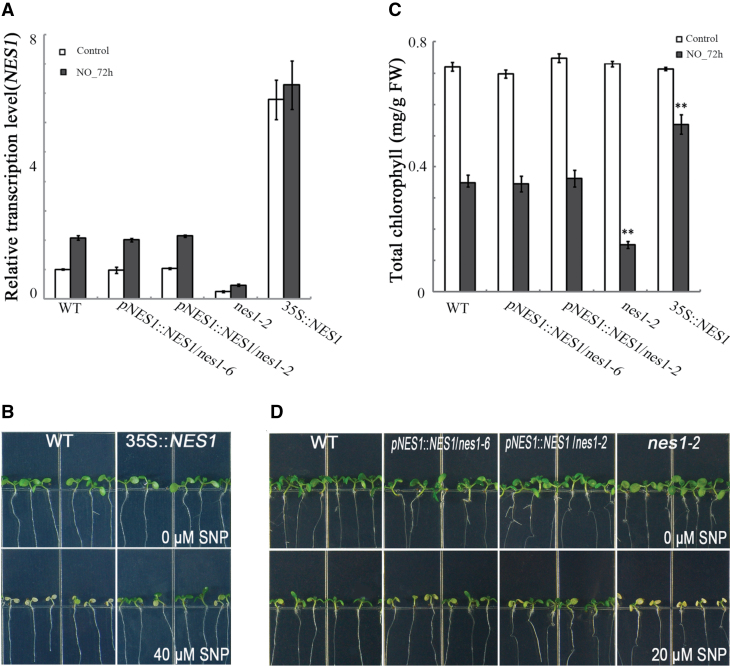
*NES1* as a repressor in nitric oxide-induced cotyledon senescence. (A) Relative transcriptional quantification of *NES1* in transgenic plants. Seedlings treated as in Fig. 1E. (B) The *NES1-*overexpressing seedlings (35S::*NES1*) treated with 40 μM nitroprusside. (C) Chlorophyll content at 72h of plants treated as in Fig. 1A. Data are mean values of 20 seedlings ±SE, with three replicates for each sample. (D) Complementation of the transgenic plants treated as in Fig. 1A. Asterisks indicate significant differences (*P < 0.05; **P ≤ 0.01).

To assay further the function of *NES1* in nitric oxide-induced cotyledon senescence, *NES1*-overexpressing transgenic lines were generated by transformation of 35S::*NES1*. Three transgenic lines were obtained and tested, and only the strongest line was used in the experiment. In this line, the *NES1* message level was almost 10 times higher than in the wild type, but was not further induced by nitroprusside (Fig. 3A). When the line was exposed to as much as 40 μM nitroprusside, the cotyledons remained green whereas those of the wild type appeared strongly bleached (Fig. 3B). The visual impression was confirmed by measuring the cotyledon chlorophyll content 72h after 20 μM nitroprusside treatment (Fig. 3C). The overexpression lines, driven by the 35S promoter, expressed the coding sequence of *NES1*. To determine whether this led to artefacts, the coding sequence from the native promoter (*pNES1::NES1*) was expressed and introduced into either the *nes1-6* or the *nes1-2* background. These lines were indistinguishable from the wild type in terms of mRNA level (Fig. 3A), chlorophyll content (Fig. 3C), and overall appearance (Fig. 3D), thereby validating the use of the coding sequence in the overexpression lines. These results are consistent with *NES1* being a repressor of nitric oxide-induced cotyledon senescence.

### *ORE1* positively regulates nitric oxide-induced cotyledon senescence

**Fig. 4.**
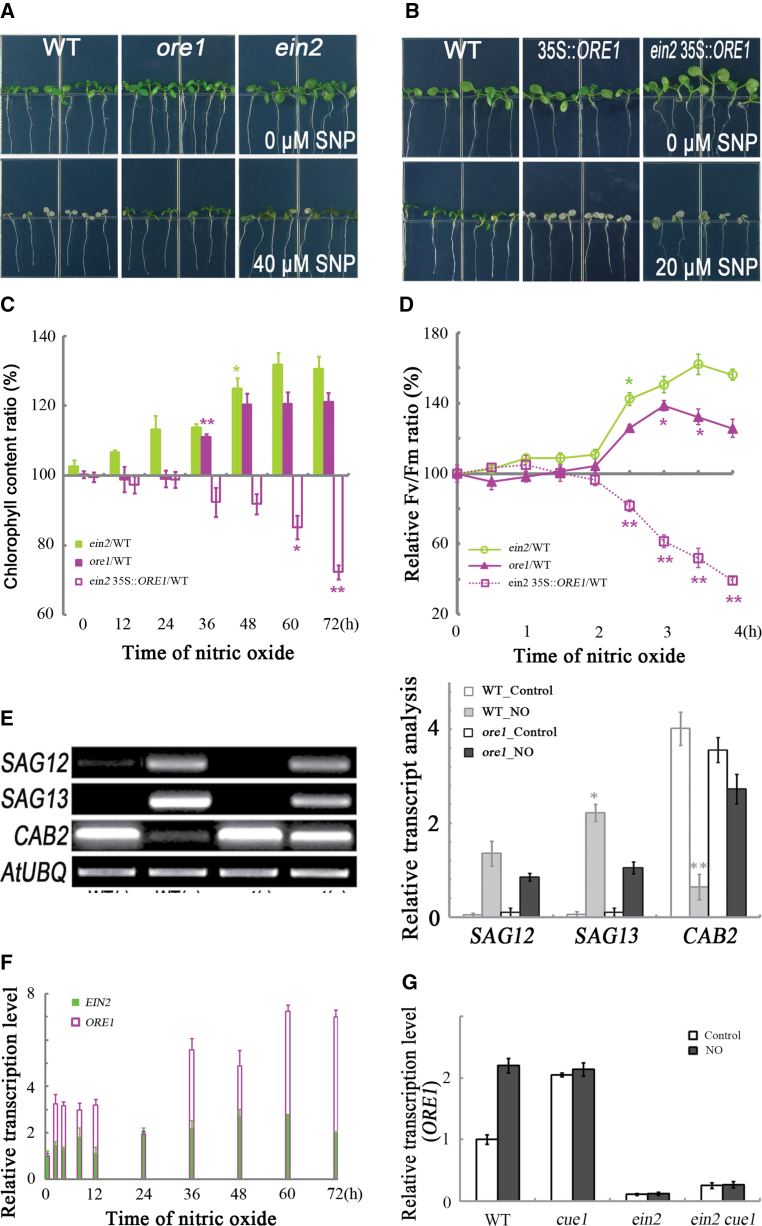
*EIN2* is required for nitric oxide to induce *ORE1* during cotyledon senescence. (A) The *ein2* and *ore1* seedlings were treated as in Fig. 3B. (B) *ORE1*-overexpressing seedlings (35S::*ORE1* and *ein2* 35S::*ORE1*) treated as in Fig. 1A. (C) Chlorophyll content as a function of time for plants treated as in (B). Data are the means of 20 seedlings ±SE, with four replicates for each time point. (D) Chlorophyll fluorescence parameter (*F*_v_/*F*_m_) for plants treated with nitric oxide. Data are means of five seedlings ±SE, with four replicates for each time point. (E) Semi-quantitative RT-PCR analysis of the indicated genes for plants treated as in Fig. 1E. The intensity of bands was statistically analysed. Data are mean values ±SE, with three replicates for each sample. (F) The *EIN2* and *ORE1* transcript abundance as a function of treatment time. Seedlings treated as in Fig. 1B. (G) The *ORE1* expression in *cue1* and *ein2*. Seedlings treated as in Fig. 1E. Asterisks indicate significant differences (*P < 0.05; **P ≤ 0.01).

During developmental senescence in *Arabidopsis* leaves, the increasing expression of the transcription factor *ORE1* acts as a key positive regulator in inducing senescence-associated downstream genes (Rauf *et al.*, 2013). To explore the role of *ORE1* in nitric oxide-regulated cotyledon senescence, the response to nitric oxide in *ore1* was checked and it was found that nitric oxide-induced cotyledon senescence was delayed in the mutant (Fig. 4A). Conversely, transgenic plants overexpressing *ORE1* in the wild-type background (35S::*ORE1*) had accelerated cotyledon senescence under nitric oxide treatment (Fig. 4B). These phenotypes were confirmed by measuring the cotyledon chlorophyll content and *F*_v_/*F*_m_ ratio (Fig. 4C, D). Finally, in *ore1*, nitric oxide treatment changed the levels of *SAG12*, *SAG13*, and *CAB2* to a lesser extent than in the wild type (Fig. 4E).

*ORE1* induction requires *EIN2* during developmental leaf senescence (Kim *et al.*, 2009). Therefore, experiments were conducted to determine whether this is also true for nitric oxide-induced senescence using the cotyledon system. First, the phenotype of *ein2* was checked to determine that it was nitric oxide-mediated late cotyledon senescence (Fig. 4A). Transgenic plants overexpressing *ORE1* in the *ein2* background were then found to present early cotyledon senescence under nitric oxide treatment (Fig. 4B). When chlorophyll (Fig. 4C) and the *F*_v_/*F*_m_ ratio were quantified (Fig. 4D), *ein2* was affected more strongly than *ore1*. After nitric oxide treatment, the chlorophyll content decreased in the wild type (Fig. 1C). In *ein2* and *ore1* mutants, the percentage of chlorophyll and the *F*_v_/*F*_m_ ratio rose linearly with respect to the wild type after nitric oxide treatment. This reflected that the *ein2* and *ore1* mutants prevented the loss of chlorophyll and of electron transport capacity with similar kinetics. However, the overexpression of *ORE1* in the *ein2* background reversed the phenotype of the *ein2* mutants, and caused a more rapid decline of the chlorophyll content and *F*_v_/*F*_m_ ratio, compared with the wild type, under nitric oxide treatment. This was also supported by the molecular evidence (Fig. 4F). Indeed, *ein2* essentially had a limited background level of the *ORE1* message in cotyledons. However this was not increased by nitric oxide treatment (Fig. 4G), meaning that the *ORE1* induction relied on *EIN2*. As an alternative to nitroprusside treatment, the *cue1* (CAB underexpressed) mutant was also examined, which has elevated levels of endogenous nitric oxide (He *et al.*, 2004). Consistently, in *cue1*, the *ORE1* transcript level was increased even in the absence of nitroprusside treatment, while in *cue1 ein2*, the level of *ORE1* was low and not increased by treatment. Evidently *EIN2* is required for nitric oxide to induce *ORE1*, at least in cotyledons. These results confirm the positive *EIN2*-associated *ORE1* pathway during nitric oxide-induced cotyledon senescence.

### *NES1* and *ORE1* in nitric oxide-induced cotyledon senescence

**Fig. 5.**
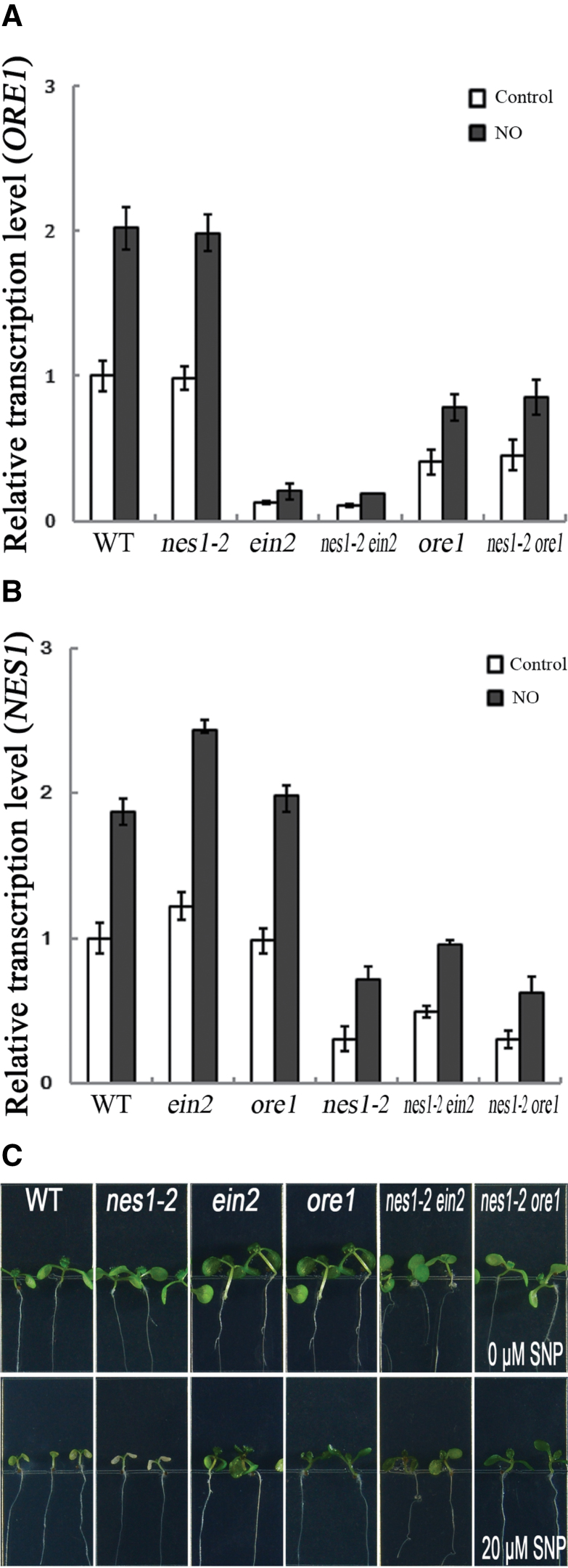
*EIN2* and *ORE1* are epistatic to *NES1* during nitric oxide-induced cotyledon senescence. (A) Relative transcriptional quantification of *ORE1* in the mutants. Seedlings treated as in Fig. 1E. Transcript quantified as in Fig. 2B. (B) The relative transcriptional quantification of *NES1*. Seedlings treated as in Fig 1E. Transcript quantified as in (A). (C) Phenotype analysis of the mutants. Seedlings treated as in Fig. 1A.

To explore the relationship between *NES1* and *ORE1* in the regulation of nitric oxide-induced cotyledon senescence, *nes1-2 ein2* and *nes1-2 ore1* double mutants were generated, and the *NES1*, *EIN2*, and *ORE1* mutants were further analysed. Nitric oxide-regulated cotyledon senescence was accelerated in the *nes1-2* mutant (Fig. 1A) and *ORE1* expression was indistinguishable in both the *nes1-2* mutant and the wild type (Fig. 5A). This implied that *NES1*, without interference from *ORE1*, was responsible for the accelerated senescence in the *nes1-2* mutant. In addition, nitric oxide-delayed cotyledon senescence in the *ore1* mutants (Fig. 4A), together with *NES1* expression indistinguishable in the *ore1* mutant and the wild type (Fig. 5B), suggested that *ORE1* mainly contributed to the delayed cotyledon senescence in the *ore1* mutant. The *NES1* transcript level was slightly higher in the *ein2* mutant than that in the *ore1* mutant and the wild type. In the *ein2* mutant, transcription of *ORE1* declined significantly (Fig. 5A) and *NES1* increased slightly (Fig. 5B) compared with that in the wild type. Alternations of these genes co-operatively contribute to the delay of nitric oxide-mediated cotyledon senescence in the *ein2* mutant (Fig. 4A).

These results illustrated that *NES1* and *ORE1* did not affect each other’s expression in either induced or delayed senescence, indicating that there may be limited direct interaction between *NES1* and the *EIN2*-associated *ORE1* pathway during nitric oxide-induced cotyledon senescence. However, the test of responses to nitric oxide in the double mutant *nes1-2 ein2* and *nes1-2 ore1* showed that both *ein2* and *ore1* were epistatic to *nes1-2*, in terms of visual inspection of cotyledon colour (Fig. 5C) and the *ORE1* transcripts (Fig. 5A). These results imply that the delayed cotyledon senescence in the *nes1-2 ein2* and *nes1-2 ore1* double mutants is mostly driven by the *EIN2*-associated *ORE1* pathway.

## Discussion

Early cotyledon senescence leads to various alterations in the physiological homeostasis of plants, which can result in plant seedlings failing to initiate and ultimately cause agricultural losses (Zhou and Brown, 2006; Chandler, 2008), but the molecular mechanism of cotyledon senescence is currently uncertain. Previous studies have focused on characterizing age-dependent cotyledon senescence in different plant species, such as soybean, cucumber, cotton, and pumpkin (Bick and Strehler, 1971; Kanazawa *et al.*, 2000; Xie *et al.*, 2008; Ananieva *et al.*, 2008*b*) and light irradiation accelerated cotyledon senescence (Watanabe *et al.*, 1994; Wilhelmová *et al.*, 2004). In this study, the phenotype of nitric oxide-induced cotyledon senescence was investigated and the *nes1* mutant was characterized as having early cotyledon senescence which was induced by nitric oxide.

Nitric oxide is claimed to serve as a biological mediator (Besson-Bard *et al.*, 2008), in a wide array of physiological processes, including senescence (Cam *et al.*, 2012). Early studies have described age-dependent senescence as being associated with a significant decrease in intrinsic nitric oxide generation (Corpas *et al.*, 2004), and *in vitro* nitric oxide fumigation was found to delay the senescence process (Mishina *et al.*, 2007). Both of these results suggest that nitric oxide acts as a negative regulator in leaf senescence. In contrast, during salt-triggered senescence, the level of endogenous nitric oxide sharply increased (David *et al.*, 2010). This suggests that the role of nitric oxide may differ in developmental and stress-induced senescence. One explanation is that the function of nitric oxide is strongly dependent on its concentration (Procházková and Wilhelmová, 2011). This explanation has been supported by a study of leaf senescence, which shows that small doses of nitric oxide delay senescence, while large doses accelerate the process (Selcukcan and Cevahir, 2008). These results emphasize that the dose-dependent action of nitric oxide is a double-edged sword (Colasanti and Suzuki, 2000).

To study the function of nitric oxide in cotyledon senescence, SNP was used to release nitric oxide. In these experimental conditions, the concentration of nitric oxide in sealed Petri plates initially undergoes complex transients and requires at least 2h to reach a stable level (Ferrero *et al.*, 1999). Therefore, the dynamic behaviour observed for the *F*_v_/*F*_m_ ratio probably reflects these dynamics. However, the sharp and significantly greater decrease in the mutant is clear (Fig. 1D).

The transcription factor *ORE1* not only initiates developmental leaf senescence, but also plays a role in stress-induced leaf senescence, such as heat stress, oxidative stress, salt stress, and drought stress (Harding *et al.*, 1990; Woo *et al.*, 2004; Valente *et al.*, 2009; Balazadeh *et al.*, 2010; Wagstaff *et al.*, 2010). All of these stresses are coupled with a massive accumulation of nitric oxide (Bouchard and Yamasaki, 2008; Lozano-Juste and León, 2010). As was shown in this study, during nitric oxide-accelerated cotyledon senescence, *EIN2* and *ORE1* play important positive roles. Therefore, during senescence triggered by an environmental stimulus, nitric oxide is probably involved in transmitting the stress signal to the *EIN2*-associated *ORE1* pathway.

Previous studies have revealed that *EIN2* functions as a positive regulator of ethylene- and dark-induced senescence (Kim *et al.*, 2009; Chen *et al*, 2012). Recently, dark-accelerated senescence in the nitric oxide-deficient mutant *nos1*/*noa1* was reverted by *EIN2* mutation (Niu and Guo, 2012), which suggests that *EIN2* is involved in nitric oxide-regulated leaf senescence. Although the mutation of *NOS1*/*NOA1* is associated with a significant reduction in nitric oxide production, the role of *NOS1*/*NOA1* in biosynthesis is still controversial (Guo *et al.*, 2003; Zemojtel *et al.*, 2006; Moreau *et al.*, 2008). The present study not only confirms the role of *EIN2* in nitric oxide-regulated senescence but also reveals the positive function of *EIN2*-associated *ORE1* in nitric oxide-induced cotyledon senescence (Fig. 4).

The transcription factor *ORE1*/*AtNAC2* affects leaf senescence under a number of conditions, including age-dependent and stress-induced senescence. *ORE1*/*AtNAC2* functions in nitric oxide-induced cotyledon senescence (Fig. 4A) and *ORE1* may extend the role in age-dependent cotyledon senescence. The *ORE1* transcript level in cotyledons was massively up-regulated by day 21 (Supplementary Fig. S4A at *JXB* online), which was approximately the time of onset of developmental senescence (Supplementary Fig. S1A). In contrast, this pronounced up-regulation of *ORE1* had not occurred in leaves by day 21 (Supplementary Fig. S4A), where leaf senescence did not start until several days later. Together, these findings suggest that *ORE1* plays a positive role in developmental cotyledon senescence. And the results of studies on *ORE1*/*AtNAC2* with different model systems are quite similar to those demonstrated in *Arabidopsis* leaves and cotyledons. For example, *RhNAC2* was found to regulate flower senescence in *Rosa hybrida* (Dai *et al.*, 2012), *MaNAC2* was shown to be involved in banana fruit ripening (Shan *et al.*, 2012), and *AtNAC2* was demonstrated to function in *Arabidopsis* silique senescence (Kunieda *et al.*, 2008). In conclusion, *ORE1*/*AtNAC2* acts as a key element in positively regulating whole-plant senescence during age-dependent and induced processes.

Besides the positive role of the *EIN2*-associated *ORE1* regulatory pathway during senescence, negative regulation by *NES1* plays an important role in the nitric oxide-induced cotyledon senescence process. During the development process, *NES1* has little impact on age-dependent cotyledon senescence, as the *nes1-2* mutants and 35S::*NES1* transgenic plants showed similar developmental stages to the wild type. At the same time, just as the level of *ORE1* rises in senescing cotyledons, *NES1* was able to be gradually induced during the natural cotyledon ageing process. However, the patterns were quite different in leaves. The level of *ORE1* only increased just before the initiation of senescence in cotyledons (Supplementary Fig. S4A at *JXB* online) and true leaves (Kim *et al.*, 2009), compared with *NES1*, which was generally induced in both cotyledons and true leaves. Once *NES1* was induced in senescent cotyledons, the up-regulation pattern was consequently observed in young leaves (Supplementary Fig. S4B). The gradual increase of *NES1* in cotyledon developmental ageing implies that *NES1* is affected during developmental cotyledon senescence. The consequent induction of *NES1* in young leaves suggests that *NES1* might have another function in true leaf development. NES1 was also identified as nuclear-located MAD1, which interacted with MAD2 to suppress premature cell division at the *Arabidopsis* root meristem (Ding *et al.*, 2012). However, the accelerated senescence in the *nes1-2* mutant and the nitric oxide-induced *NES1* expression pattern are obvious (Figs 1A, 2C). In conclusion, *NES1* negatively regulates nitric oxide-accelerated cotyledon senescence, but not developmental senescence in cotyledons.

During nitric oxide-triggered cotyledon senescence, *ORE1* and *NES1* are both induced (Figs 2C, 4F), and function antagonistically against each other. The interaction between them reveals two distinct profiles during induced cotyledon senescence. The *EIN2*-associated *ORE1* is a senescent signal transducer, and the induced *NES1* functions as a negative regulator modulating the process, resulting from the evolution and maintenance in accordance with rigorous controlled plant acclimation (Heil and Baldwin, 2002). The nitric oxide-accelerated cotyledon senescence in *nes1-2* and delayed cotyledon senescence in 35S::*NES1* revealed its dominant role in repression of senescence. In 35S::*NES1*, although ectopically overexpressed, the delayed senescence phenotype (Fig. 3B) was due to the large increase of *NES1* (Fig 3A), which indicates that *NES1* could be epistatic to *ORE1*, in a dose-dependent manner. Together with the finding that *ORE1* was epistatic to *NES1* in nitric oxide-induced cotyledon senescence (Fig. 5C), this shows the balance of *ORE1* and *NES1* might be a critical factor in determining the fate of plant cotyledons. Once *NES1* was dominant, plant senescence would be postponed, even without the initiation of senescence.

## Supplementary data

Supplementary data are available at *JXB* online.

Figure S1. Age-dependent cotyledon senescence.

Figure S2. Nitric oxide induces cotyledon senescence.

Figure S3. The fine genetic and physical map-based cloning of *NES1*.

Figure S4. The transcription level of *ORE1* and *NES1* during cotyledon and leaf development.

Table S1. Genetic analysis of allelic *NES1*.

Table S2. Primers for semi-quantitative RT-PCR.

Table S3. Primers for quantitative real-time PCR.

Supplementary Data
