# Supplementary material for: Nitric oxide induces cotyledon senescence involving co-operation of the NES1/MAD1 and EIN2-associated ORE1 signalling pathways in Arabidopsis
Source: J Exp Bot. 2013 Dec 12;65(14):4051–63. doi: 10.1093/jxb/ert429 (PMC4106434; doi:10.1093/jxb/ert429)
Supplement: Supplementary Data [file supp_65_14_4051__index.html]

Nitric oxide induces cotyledon senescence involving co-operation of the NES1/MAD1 and EIN2-associated ORE1 signalling pathways in Arabidopsis — Nitric oxide induces cotyledon senescence involving co-operation of the NES1/MAD1 and EIN2-associated ORE1 signalling pathways in Arabidopsis — Supplementary Data 

# Nitric oxide induces cotyledon senescence involving co-operation of the *NES1*/*MAD1* and *EIN2*-associated *ORE1* signalling pathways in *Arabidopsis*

## Supplementary Data

Data files

**Files in this Data Supplement:**

- Supplementary Data - Supplementary Data
