# Supplementary material for: Nitric oxide induces cotyledon senescence involving co-operation of the NES1/MAD1 and EIN2-associated ORE1 signalling pathways in Arabidopsis
Source: J Exp Bot. 2013 Dec 12;65(14):4051–63. doi: 10.1093/jxb/ert429 (PMC4106434; doi:10.1093/jxb/ert429)
Supplement: Supplementary Data [file supp_ert429_jexbot109918_file001.pdf]

Supplementary data

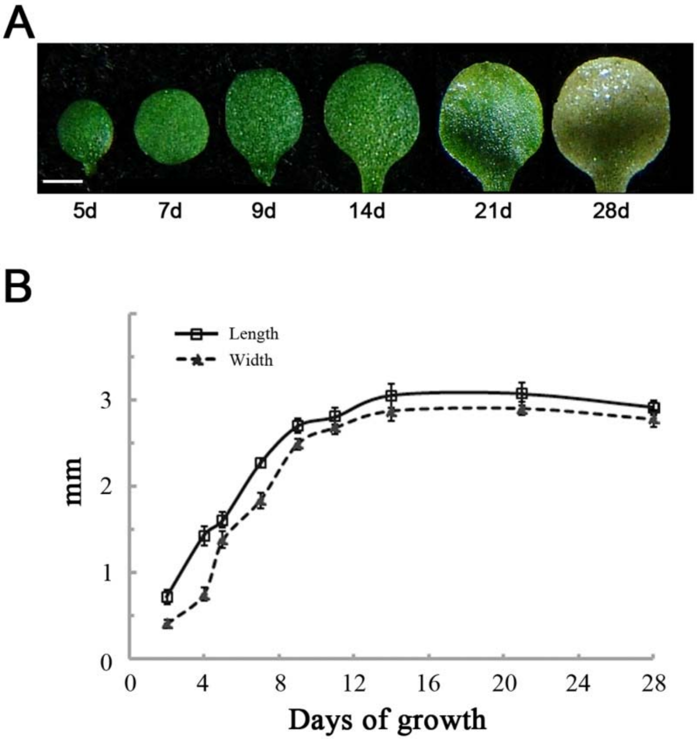

Figure 1. Age-dependent cotyledon senescence process.

(A) Developmental cotyledon senescence in the wild type, Bar= 1mm.

(B) Cotyledon width and length in the wild type during development.

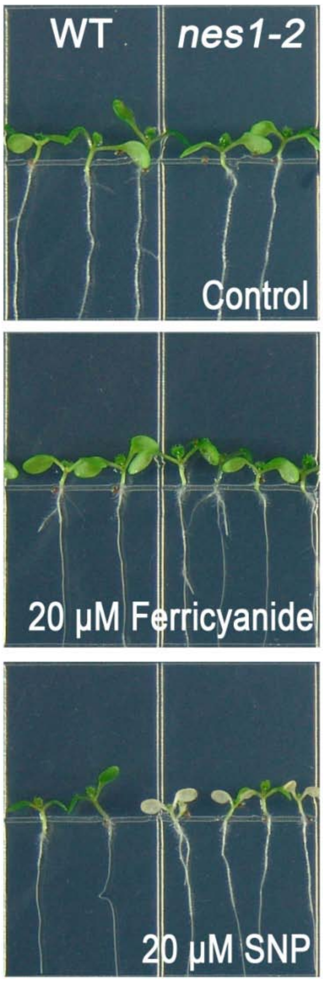

Figure 2. Nitric oxide induces cotyledon senescence

Phenotype comparison of the 5-day-old wild type (WT) and *nes1-2* treated as indicated for 72 hours.

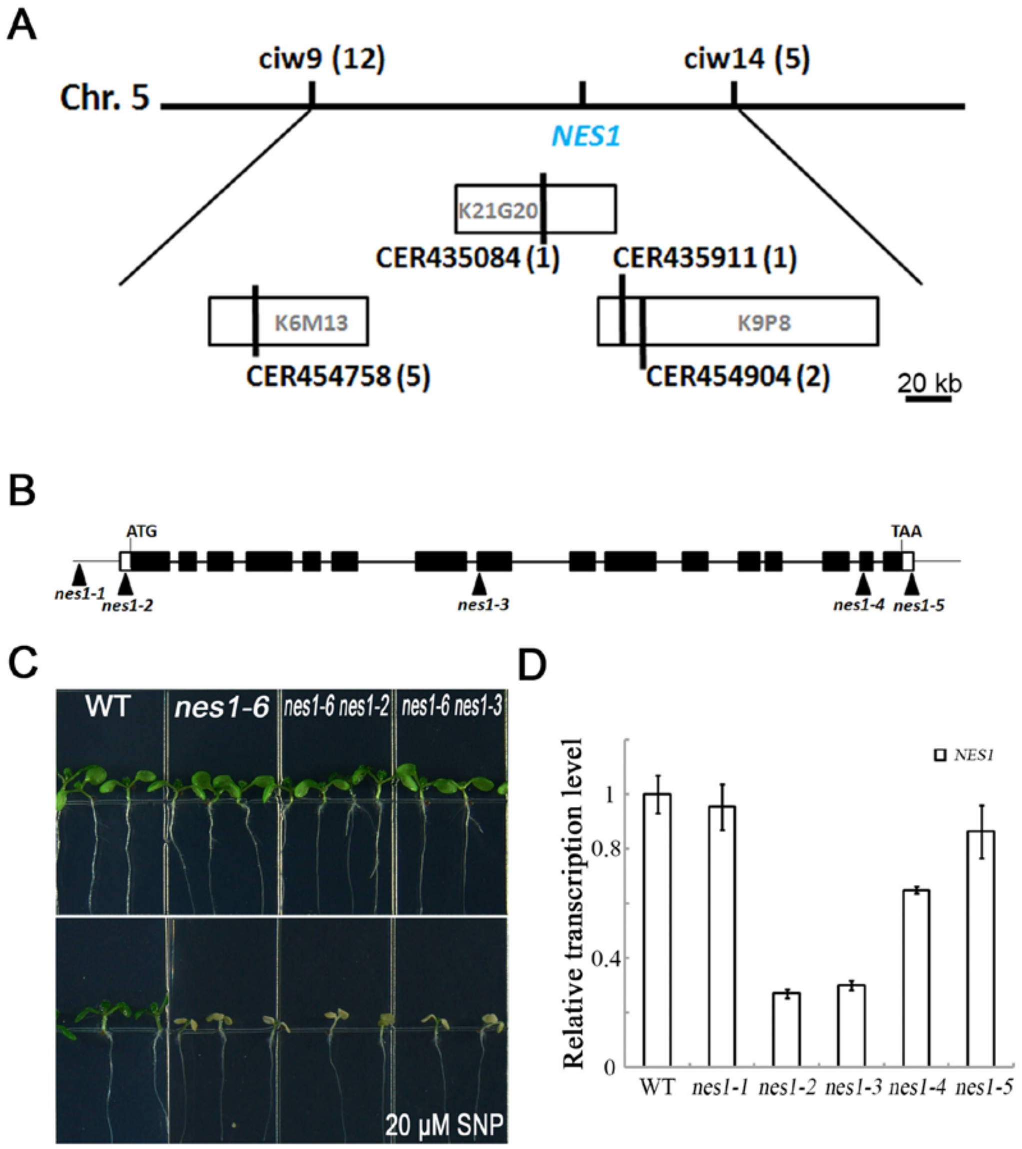

Figure 3. The fine genetic and physical map-based cloning of *NES1*.

(A) Fine genetic and physical mapping of *NES1*. **(B)** T-DNA insertion locations of the five *nes1* mutants. **(C)** Phenotype analyses of F1 generation: *nes1-6* cross with *nes1-2* and *nes1-3* separately. Plants treated with 20 μM nitroprusside for 72 hours. **(D)** The transcription levels of the five *nes1* mutants, with quantitative real-time PCR analysis.

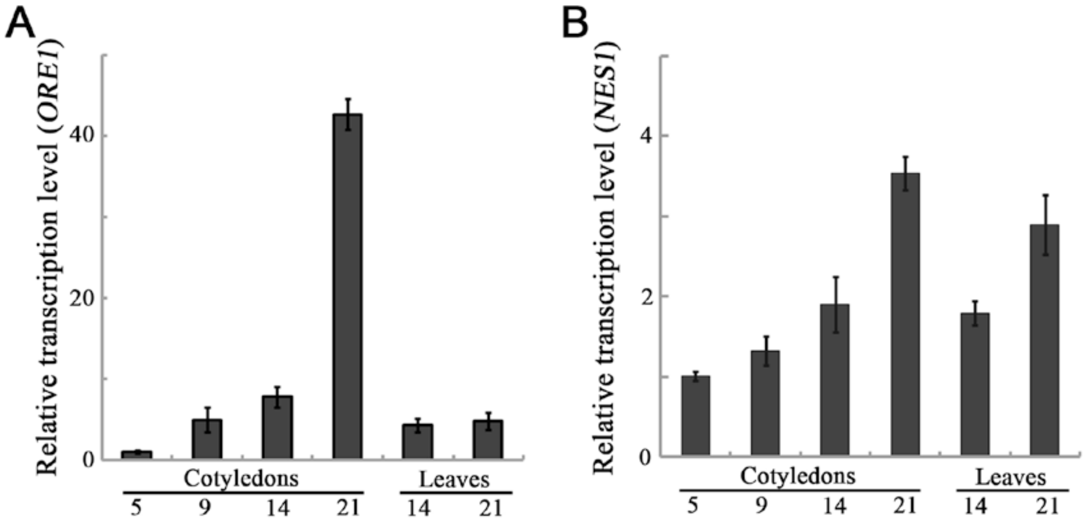

Figure 4. The transcription level of *ORE1* and *NES1* during cotyledon and leaf development.

(A) Comparison of the *ORE1* expression in cotyledons and leaves in the wild type. Transcript quantified as in Fig. S2D. **(B)** The *NES1* expression in cotyledons and leaves in the wild type. Transcript quantified as in Fig. S2D.

Table 1. Genetic analysis of allelic *NES1*.

**F0**		**F1 progeny**	**F_2 progeny**	
Insensitive to NO	Sensitive to NO	Insensitive	Insensitive	Sensitive
WT (*NN*)	*nes1-6* (*nn*)	*Nn* (17)	*NN*, *Nn* (886)	*Nn* (301)

Phenotypic ratio in F_2 generation: (886:301=2.94:1≈3:1)

Table 2. Primers for semi-quantitative RT-PCR.

**Gene**	**Locus**	**Name**	**Primer sequence**	**PCR condition temperature/cycles**
AtUBQ	AT5G25760	AtUBQ -F	CAAGAGCGCGACTGTTTAAA	58℃ / 23
		AtUBQ -R	CATTTGTGCCATTGAATTGAAC	
SAG12	AT5G45890	SAG12-F	TTTCAAAGGTGTCTCGGCATTA	60℃ / 29
		SAG12-R	GCCGTATCCAATCGCAGTTA	
SAG13	AT2G29350	SAG13-F	TCCTCTGCTGCTGGAGTCGTG	62℃ / 29
		SAG13-R	GCCGTCAACGCAAATGGTCT	
CAB2	AT1G29920	CAB2-F	TGGAGACTACGGATGGGACA	58℃ / 23
		CAB2-R	GTGACGATGGCTTGAACGAA	

Table 3. Primers for quantitative real-time PCR.

Gene	Locus	Primer name	Primer sequence
ACTⅡ	At3g18780	ACTⅡ-F	GGTAACATTGTGCTCAGTGGTGG
		ACTⅡ-R	AACGACCTTAATCTTCATGCTGC
ORE1	At5g39610	ORE1-F	CACCAGGATTCAGATTTCAC
		ORE1-R	TCTCCCATCTTAGCCTTCC
EIN2	At5g03280	EIN2-F	CACGATGCCTTGTCACTA
		EIN2-R	CAAGCGGGTATTTCTATCT
NES1	At5g49880	NES1-F	CTTGAAAGGATGCAATGTAGG
		NES1-R	CTCCGAAGTGAATGACGATAG
